# Sanitation facilities, hygienic conditions, and prevalence of acute diarrhea among under-five children in slums of Addis Ababa, Ethiopia: Baseline survey of a longitudinal study

**DOI:** 10.1371/journal.pone.0182783

**Published:** 2017-08-30

**Authors:** Metadel Adane, Bezatu Mengistie, Helmut Kloos, Girmay Medhin, Worku Mulat

**Affiliations:** 1 Ethiopian Institute of Water Resources (EIWR), Addis Ababa University, Addis Ababa, Ethiopia; 2 College of Health and Medical Sciences, Haramaya University, Haramaya, Ethiopia; 3 Department of Epidemiology and Biostatistics, University of California, San Francisco, United States of America; 4 Aklilu Lemma Institute of Pathobiology, Addis Ababa University, Addis Ababa, Ethiopia; 5 Department of Civil and Environmental Engineering, University of Connecticut, Storrs, United States of America; University of Otago, NEW ZEALAND

## Abstract

**Background:**

In developing countries, children under the age of five years who live in slums are highly vulnerable to diarrhea. However, there is a paucity of information on the relationship between sanitation facilities and hygienic conditions to acute diarrhea among under-five children in slum areas of Addis Ababa, Ethiopia. Therefore, this study examines the sanitation facilities and hygienic conditions in the slums of Addis Ababa and identifies the main factors significantly associated with acute diarrhea among children aged 0–50 months in those slums.

**Methods:**

A community-based cross-sectional household survey was carried out between September and November 2014, that then served as the baseline survey of a longitudinal study. For this survey, 697 children aged 0–50 months were recruited from two slum districts in Addis Ababa. A pre-tested structured questionnaire and an observational checklist were used for data collection. Multivariable logistic regression analysis was used to identify sanitation facilities and hygiene-related factors that were significantly associated with acute diarrhea by controlling potential confounding effects of selected socio-demographic factors. Adjusted odds ratio (AOR) with corresponding 95% confidence interval (CI) was used to quantify the strength of association.

**Main findings:**

The prevalence of acute diarrhea among children aged 0–50 months in the study area was 11.9% and 94.6% of the sanitation facilities were unimproved. Sharing of a sanitation facility by six or more households (AOR = 4.7; 95% CI: 2.4–9.4), proximity of sanitation facilities within 15 meters of homes (AOR = 6.6; 95% CI: 2.5–17.0), presence of feces (AOR = 3.9; 95% CI: 1.5–10.3) and flies (AOR = 2.5; 95% CI: 1.3–5.0) on the floor of and/or around sanitation facilities, and presence of uncollected garbage inside house compounds (AOR = 3.2; 95% CI: 1.2–8.4) were significantly associated with acute diarrhea.

**Conclusion:**

This study reveals the slum environment to be high risk for diarrhea due to close proximity of sanitation facilities to homes, sharing of sanitation facilities, and poor hygiene of the sanitation facilities and housing compounds. We recommend the development of a comprehensive diarrheal disease prevention program that focuses on improving the cleanliness of the sanitation facilities and housing compounds. Increasing the number of improved sanitation facilities at an appropriate distance from houses is also essential in order to reduce the number of households that share one latrine.

## Background

The United Nations Human Settlements Programme has reported massive urban growth in low- and middle-income countries resulting in sprawling slums that are now home to more than half the population of cities such as Mumbai in India; Kibera slum in Nairobi, Kenya; Mexico City in Mexico [1]; and Addis Ababa in Ethiopia [2]. The rapidity of urbanization in these countries has caused dynamic growth of urban slums and contributed to increasing numbers of informal slum dwellers [3, 4]. The result has been overcrowded living conditions [5]; inadequate sanitation facilities [6]; and exposure of slum dwellers, especially children under five years of age, to a high risk of disease [7]. Worldwide, about eight million children died in 2010 before reaching the age of five, mainly due to poor sanitation facilities and unhygienic conditions [8].

Most people expect that urban areas have better child health and lower child mortality than rural areas [9, 10]. However, recent studies have consistently indicated that under-five children in cities of developing countries have been frequent victims of diarrhea, mainly due to lack of improved sanitation facilities, poor hygienic practices [11], and the low hygienic status of shared sanitation facilities [12]. Despite the overall favorable health statistics in urban areas, several studies have pointed out large variations among countries and within urban areas and the potential influence of slums on these variations [9, 13–15]. Disparities in health determinants between slum and non-slum areas have varied within the socioeconomic context of each country [15]. For example, researchers have found that urban caregivers in Ethiopia disposed of the feces of under-five children more safely than did rural caregivers and safe disposal was associated with having an improved sanitation facility [16]. However, the mere presence of household sanitation facilities in urban areas did not necessarily result in favorable health outcomes [17]. Thus, policies based on the current system of monitoring sanitation facilities fail to consider the ranges of challenges and solutions in meeting sanitation needs [18].

Slums are known in Ethiopia as “*yedekemu betoch/seferoch*,” meaning deteriorated/dilapidated houses or settlements [19]. This definition of slums focuses on their physical structure without considering their socio-economic and health characteristics, both of which must also be addressed for remedial and preventive actions to succeed. According to Sclar et al. [20], national governments and global society in general could accumulate a massive health debt if countries neglect the health of children in urban slums. In 2011, the Demographic and Health Survey data revealed that diarrhea prevalence for under-five children in Addis Ababa was 9.4% [21]; the survey did not investigate sanitation facilities and hygiene-related factors associated with diarrhea. Recent studies in various developing countries have recommended that particular attention be given to examining health determinants for slum-dwelling children underfive [7, 22]. Furthermore, in the slums of Addis Ababa, to our knowledge, no other studies have been undertaken on sanitation facilities and hygienic conditions as factors associated with acute diarrhea. Lack of reliable data, particularly on sanitation facilities and hygienic conditions in slum areas of Addis Ababa, hinders planning for and implementation of diarrhea prevention programs among under-five children. Effective diarrhea prevention programs may facilitate the achievement of UN Sustainable Development Goals by 2030, specifically Goal 3 (ensure health and well-being for all, at every stage of life), particularly Target 3.2 (end preventable deaths of children under five years of age, with all countries aiming to reduce under-five mortality to at least as low as 25 per 1,000 live births) and Goal 6 (ensure availability and sustainable management of water and sanitation for all), particularly Target 6.2 (achieve access to adequate and equitable sanitation and hygiene for all, and end open defecation) [23].

Therefore, this study was designed to examine the sanitation facilities and hygiene practices in the slums of Addis Ababa in relation to acute diarrhea among under-five children. Results may help urban health policy makers and program managers in the development and implementation of improved sanitation facilities and hygiene programs for preventing acute diarrhea in the slum areas of Addis Ababa and other slums in Ethiopia and throughout sub-Saharan Africa.

## Materials and methods

### Study setting

**Fig 1:**
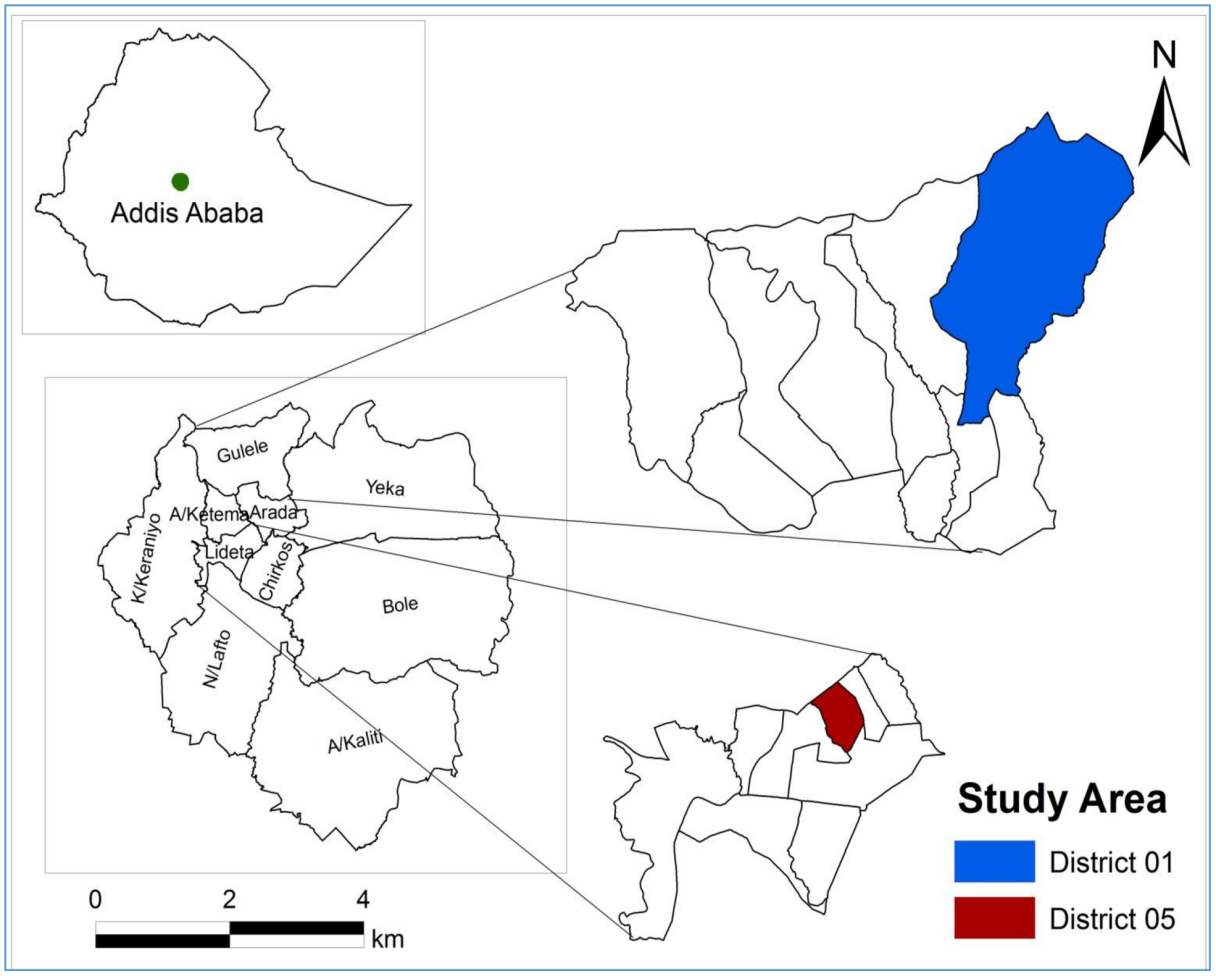
Map of the study area.

This study was conducted in two slum districts (*woredas*) in Addis Ababa: Gullele Sub-City’s District 01 and Lideta Sub-City’s District 05 (Fig 1). Addis Ababa’s population was estimated to be 3,273,000 in 2014–15, of which 1,551,000 (47.4%) were males and 1,722,000 (52.6%) were females [24]. In 2008, the city-wide data on basic indicators in Addis Ababa showed that 26% of the houses and the majority of slum dwellers had no toilet facilities, 33% of households shared a toilet with more than six households, 35% of the generated garbage/refuse was never collected, and 71% of the households did not have adequate sanitation facilities [25].

### Study design and outcome variable

A community-based cross-sectional study design was used to gather data regarding children aged 0–50 months in the slums of Addis Ababa between September and November 2014. Children over 50 months were not included because the study was a baseline survey in a longitudinal study consisting of four rounds of surveys to be conducted at three-month intervals among the same study participants. During recruitment of children for the baseline survey, one of the criteria was that all study participants should be under five years of age at the end of the longitudinal study, at the fourth round, in August 2015. Therefore, the age ranges of participants at each round were as follows: First round, 0–50 months; second round, 3–53 months; third round, 6–56 months; and fourth round, 9–59 months. Thus, for the baseline survey, we used the age category of 0–50 months.

The outcome variable of this study was acute diarrhea, denoted as yes (1) or no (0); where yes indicated the presence of acute diarrhea and no indicated the absence of acute diarrhea during the two weeks prior to the baseline survey. Using the outcome variable of presence of acute diarrhea, we estimated the prevalence of acute diarrhea among the participating children.

### Sample size

The sample size estimation for this study was based on the fact that the study was the baseline survey of a longitudinal study; sample size was calculated with the primary objective of studying the seasonal prevalence of acute diarrhea among under-five children. However, since the longitudinal study utilized a repeated cross-sectional survey through follow-up of the same study participants, the sample size estimation methods and assumptions for calculating the same sample size were the same. Hence, sample size was estimated using the single-proportion formula [26]: *n* = (*Z*_1_-_*a*/2_)^2^*P(1-*P*)/*W*^2^ with the assumptions that *Z*_1_-_*a*/2_ is 95% CI, *W* has a margin of error of 3%, and *P* is 11% estimated prevalence of acute diarrhea among under-five children in the slums of Addis Ababa. The calculated sample size was 418. Considering a design effect of 1.5 and a 10% non-response rate, a final sample size of 697 children was determined.

### Study population and sampling procedures

A multi-stage sampling method with two stages was employed to select the study population. In the first stage, two slum districts were randomly selected from the identified slum districts in Addis Ababa. During this stage, slum districts included in the city-wide slum upgrading plan (being implemented until 2025 by the Addis Ababa City Administration Housing Agency) were excluded from the study. To determine the sampling population in the study districts, a preliminary survey was conducted in the two selected slum districts through transect walks in a house-to-house enumeration of children whose maximum age would be 50 months at the end of the baseline survey period. Then, sample sizes were proportionally allocated for the two districts. By the second stage, systematic sampling techniques were used in every third household to select study participants. Households where study participants were not available during the survey were revisited once on the same day or the next day. If not available again, the study participant was considered a non-respondent.

### Inclusion and exclusion criteria

Children aged 0–50 months were included during selection of the study participants. In households with more than one eligible child, one child was randomly selected and recruited into the study. Eligible children with bloody diarrhea and/or persistent diarrhea two weeks prior to the survey were excluded. Bloody diarrhea was excluded because it is frequently caused by dysentery, and persistent diarrhea was excluded because of its duration of 14 days or longer [27]. Both of these conditions were beyond the scope of this study.

### Operational definitions

#### Acute diarrhea

Diarrhea was identified using World Health Organization (WHO) [28] signs and symptoms for diarrhea by questioning the participants’ caregivers about signs and symptoms of diarrhea two weeks prior to data enumeration, such as consistency of bowel movements, fever, vomiting, blood in stool, mucus in stool, watery stool, and frequency of abnormal stool. The WHO protocol [28] defines diarrhea as the passage of three or more abnormally loose, watery, or liquid stools over a 24-hour period. However, the WHO protocol did not specify the recall period and the types of diarrhea (acute/watery diarrhea, bloody diarrhea, or persistent diarrhea). Because our study focused on acute diarrhea, we adopted a two-week recall period, as specified in the World Gastroenterology Organization global guidelines for acute diarrhea surveys [29].

#### Sanitation

WHO defines sanitation as the provision of facilities and services for the safe disposal of human feces and urine. Sanitation also refers to the maintenance of cleanliness (hygienic conditions) through services such as proper garbage collection and wastewater disposal.

#### Sanitation facilities

Refers to latrines of various types.

#### Shared sanitation

Refers to unimproved sanitation facilities that are shared by two or more households, including public latrines.

#### Improved sanitation

The Joint Monitoring Programme (JMP) for water supply and sanitation of WHO and UNICEF defines improved sanitation as flush toilets and pit latrines using the flush/pour-flush method that are connected to either a sewer or a septic system, ventilated improved pit latrines, and pit latrines with slab and composting toilet [30]

#### Unimproved sanitation

The JMP for water supply and sanitation of WHO and UNICEF defines unimproved sanitation as pit latrines without a slab, open defecation, and public latrines. Improved sanitation facilities that are shared by two or more households are classified as unimproved because shared sanitation facilities tend to be less hygienic and less accessible than private sanitation facilities used by a single household [30].

#### Open defecation

In this study, open defecation is a self-reported behavior, including defecating in fields, bushes, forests, open bodies of water, or other open spaces.

#### Houses rented from government and private owners

Houses rented from the government are affordable for low-income citizens, whereas houses rented from private owners are expensive. However, renting private houses did not reflect a higher household income or socio-economic variation; it merely indicated a relatively larger expense.

### Ethical considerations

Ethical clearance for this study was obtained from the Institutional Ethical Review Committee of Wollo University, College of Medicine and Health Sciences. The committee provided approval after reviewing both the protocol and the consent forms. Permission to conduct the study was obtained from Addis Ababa City Health Administration Bureau, Gullele and Lideta Sub-City health offices, and the respective study area slum district health offices. Written informed assent and consent were obtained from the caregivers of participating children, assent on behalf of the participating children and consent for the caregivers themselves. Confidentiality was assured by collecting the data anonymously and coding the names of the respondents.

### Data collection and data quality assurance

Household survey data were collected using a pre-tested structured questionnaire and an observational checklist. The questionnaire and the observational checklist were first prepared in English and then translated into Amharic for participating household use. The questionnaire was pre-tested on 10% of the study households in one randomly selected nearby slum district to evaluate face validity and to ensure that the caregivers understood the questions. Any amendment made in the questionnaire was based on the pre-test. Seven trained female nurses and environmental health professionals administered the survey by interviewing primary caregivers (mothers) using a pre-tested structured questionnaire. To reduce bias, the data enumerators were blinded and did not know if they were surveying study areas in slums or non-slums.

Data enumerators and study respondents were also blinded about the hypothesis of the study to reduce bias. Daily supervision was provided by two public health professionals and the principal investigator checking the completeness of the questionnaires and the consistency of the data. Data were entered using EpiData Version 3.1 (EpiData Association, Odense, Denmark) statistical software and then exported to the Statistical Package for the Social Sciences (SPSS) Version 24.0 (IBM Corp., Armonk, N.Y., USA) for data cleaning. In order to verify the accuracy of data entries, two generic data verification strategies were employed as described elsewhere [31].

### Independent variables

Nine socio-demographic and child-related variables were considered in this analysis as potential confounders (Table 1). Ten selected sanitation facilities and hygiene variables were also included in this study. One sanitation variable was self-reported (number of households sharing a latrine), one sanitation variable was measured by data enumerators (proximity of latrines to home), and sanitation status (improved or unimproved) was classified by the researcher based on the types of sanitation facilities reported and the number of households sharing a latrine. Types of sanitation facilities were measured by data enumerators using both self-report (open defecation) and direct observation (public latrine, pit latrine with slab, and pit latrine without slab). Four hygiene variables were directly observed by data enumerators (feces and flies on the floor and/or around the sanitation facilities, garbage/refuse and domestic sewage inside the housing compound), and two waste management variables were self-reported (garbage/refuse disposal methods and domestic sewage discharge methods) (Table 1).

**Table 1:** Description of socio-demographic, sanitation facility, and hygiene variables included in this analysis.

Variable description	Format for analysis
**Socio-demographic characteristic**	
Slum district	Binary, reference category was Gullele Sub-City’s District 01
Age of caregivers (years)	Categorical variable, reference category was caregivers’ age above 34 years
Caregivers’ educational attainment	Binary, reference category was literate caregivers. Literacy denoted as caregivers able to read and write by having attended either formal or informal education; illiteracy refers to caregivers being unable to read or write. Educational attainment was measured by self-reporting without literacy test.
Marital status of caregivers	Categorical, reference category was caregivers who were married.
Household monthly income	Binary, reference category was monthly household income $50 US^+^ or above
House ownership	Categorical variable, reference category was owned or other houses. Other houses are houses that were illegally constructed and had no owner or houses temporarily provided by families to relatives or other persons.
Household size	Binary, reference category was households with six persons or more.
Child’s age	Categorical variable, reference category was child’s age between 36 and 50 months.
Child’s sex	Binary, reference category was female sex.
**Sanitation facility variables**	
Sanitation status	Binary, reference category was improved sanitation.
Number of households sharing one sanitation facility	Binary, reference category was 1–5 households. Sharing sanitation facilities did not include households that practiced open defecation.
Types of sanitation facility used	Categorical variable, reference category was pit latrine with slab.
Proximity of sanitation facility to home	Binary, reference category was distance of sanitation facilities 15 meters or more from homes. Proximity was not measured for households that practiced open defecation.
**Hygiene variables**	
Feces observed on the floor and/or around the sanitation facilities	Binary, reference category was no feces observed on the floor and/or around the sanitation facilities during the two weeks prior to the survey.
Flies observed on the floor and/or around the sanitation facilities	Binary, reference category was no flies observed on the floor and/or around the sanitation facilities during the two weeks prior to the survey.
Uncollected garbage/refuse observed inside the house compound	Binary, reference category was uncollected garbage/refuse observed inside the house compound during the two weeks prior to the survey.
Domestic sewage observed inside the house compound	Binary, reference category was domestic sewage observed inside the house compound during the two weeks prior to the survey.
**Waste disposal method**	
Garbage/refuse disposal methods	Categorical variable, reference category was garbage/refuse disposed of through house-to-house garbage/refuse collectors or put into municipal garbage/refuse container.
Domestic sewage discharge methods	Categorical variable, reference category was domestic sewage discharged through wastewater disposal through mesh wire.

^+^The average exchange rate $1 US (United States Dollars) = 20.0 ETB (Ethiopia birr) from September to November, 2014.

### Data analysis

Data were analyzed using STATA Version 14.0 (StataCorp LP, College Station, TX). Descriptive statistics were calculated including means and ±SD (standard deviations) for continuous variables. Data analysis was performed using a binary logistic regression model at 95% CI. The modeling strategy involved estimating the crude odds ratio (OR) using bivariate analysis and adjusted odds ratio (AOR) using multivariable analysis.

Bivariate analysis was employed to identify factors associated with acute diarrhea at *p* < 0.05 without controlling confounders, whereas in the multivariable analysis, the association between sanitation and hygiene factors with acute diarrhea was examined by controlling for potential confounders [32, 33] of socio-demographic factors. Multi-collinearity of variables was assessed by calculating the variance inflation factor. Hosmer-Lemeshow statistic was used to test the goodness-of-fit of the model [34]. The adjusted model estimated the overall effect of all variables to select the significant determinants after adjustment for confounding factors. From the adjusted analysis, variables with *p* < 0.05 were taken as statistically significant and independently associated with acute diarrhea.

## Results

### Socio-demographic characteristics

Of the 697 study participants, seven caregivers were non-respondents (1%). The prevalence of acute diarrhea was 11.9%. The majority (79.4%) of the caregivers were literate; 64.2% of slum residents lived in houses rented from their district administration and 17% rented from private owners. Almost one-third (31%) of the households had six or more persons. Characteristics of other socio-demographic factors and the results of the bivariate analysis of acute diarrhea are summarized in Table 2.

**Table 2:** Bivariate analysis of socio-demographic, sanitation facility, and hygiene factors with acute diarrhea among children aged 0–50 months in slums of Addis Ababa, Ethiopia, September to November, 2014.

Variable	Number (*n*)	Percentage (%)	Acute diarrhea (yes)	OR (95% CI) ^a^
**Socio-demographic factor**				
Slum district				
District 05	319	46.2	45	1.5(0.9–2.4)
District 01	371	53.8	37	1
Age of caregivers (years)				
< 25	106	15.4	17	1.6(0.8–3.2)
25–34	405	58.7	46	1.1(0.6–1.9)
>34	179	25.9	19	1
Caregivers’ educational attainment				
Illiterate	142	20.6	21	1.4(0.8–2.4)
Literate	548	79.4	61	1
Monthly household income				
Less than $50 US	250	36.2	48	2.8(1.8–4.5)
$50 US or above	440	63.8	34	1
House ownership				
Rented from *kebele*	443	64.2	53	1.1(0.6–2.1)
Rented from private owner	117	17.0	15	1.2(0.6–2.6)
Owned or other	130	18.8	14	1
Household size				
6 or more persons	214	31.0	41	2.5(1.6–4.0)
2–5 persons	476	69.0	41	1
Marital status of caregivers				
Single	46	6.7	10	2.3(1.1–4.8)
Widowed or divorced	69	10.0	10	1.4(0.7–2.9)
Married	575	83.3	62	1
Child’s age (months)				
0–5	41	6.0	7	2.9(1.1–7.8)
6–11	103	14.9	17	2.7(1.2–6.0)
12–23	198	28.7	30	2.5(1.2–5.0)
24–35	169	24.5	16	1.4(0.7–3.2)
36–50	179	25.9	12	1
Child’s sex				
Male	378	54.8	53	1.6(0.9–2.6)
Female	312	45.2	29	1
**Sanitation facility factors**				
Sanitation facility status				
Unimproved^¥^	653	94.6	82	ǂ
Improved	37	5.4	0	1
Type of sanitation facility				
Pit latrine without slab	185	26.8	17	0.9(0.4–1.9)
Public latrine	352	51.0	40	1.1(0.6–2.2)
Open defecation	36	5.2	13	4.9(2.0–12.2)
Pit latrine with slab	117	17.0	12	1
Number of households sharing one sanitation facility*				
6 or more households	301	46.0	52	4.1(2.3–7.3)
1–5 households	353	54.0	17	1
Proximity of sanitation facility from home (meters)*				
< 15 m	430	65.7	63	6.2(2.7–14.7)
15 m or more	224	34.3	6	1
**Hygiene factors**				
Feces observed on the floor and/or around the sanitation facilities*				
Yes	414	63.3	63	7.2(3.1–16.9)
No	240	36.7	6	1
Flies observed on the floor and/or around the sanitation facilities*				
Yes	258	39.4	48	4.1(2.4–7.0)
No	396	60.6	21	1
Uncollected garbage seen inside the house compound				
Yes	382	55.4	70	5.5(2.9–10.4)
No	308	45.6	12	1
Domestic sewage seen inside the house compound				
Yes	350	50.7	66	5.0(2.8–8.9)
No	340	49.3	16	1
**Waste disposal method**				
Garbage/refuse disposal method				
Disposed into open pit	43	6.2	10	3.0(1.4–6.4)
Discarded in open area outside the compound	87	12.6	14	1.9(0.9–3.6)
Thrown away inside compound	39	5.7	10	3.4(1.6–7.4)
Taken by house-to-house garbage collectors or put into municipal garbage container	521	75.5	48	1
Domestic sewage discharge method				
Open ditch outside the compound	462	66.9	53	1.2(0.6–2.7)
Discharged inside compound	60	8.7	12	2.4(0.9–6.2)
Discharged outside compound	84	12.2	9	1.2(0.4–3.2)
Discharged with wastewater disposal through mesh wire	84	12.2	8	1

OR, Crude odds ratio; CI, Confidence interval; $US, United States Dollars.

^a^Denotes crude odds ratio using 95% confidence interval in bivariate logistic regression analysis.

^1^ Reference category.

*Not including open-defecation-user households.

^¥^Including improved sanitation facilities shared by two or more households.

^ǂ^ Odds ratio not calculated since sanitation facility status (improved vs unimproved) was not considered for bivariate and multivariable analysis because no acute diarrhea cases occurred in households that used improved sanitation facilities.

### Characteristics of sanitation facilities and hygienic conditions

The majority (94.6%) of the sanitation facilities were unimproved. Of these unimproved facilities, 11.6% were an improved type but because the improved facilities were shared by two or more households, they were categorized as unimproved. Only 5.4% of sanitation facilities were improved and used by one household. By excluding open defecation users, 353 (54%) households shared one sanitary facility with one to five other households and 301 (46%) households shared a sanitary facility with six or more households. Feces and flies were observed on the floor and/or around 63.4% and 39.4%, respectively, of the sanitation facilities. Uncollected garbage/refuse and domestic sewage were observed inside 55.4% and 50.7% of house compounds, respectively (Table 2).

### Factors associated with acute diarrhea in multivariable analysis

The multivariable analysis shows that shared use of sanitation facilities by six or more households, proximity of sanitation facilities within 15 meters of homes, presence of feces and flies on the floor and/or around the sanitation facilities, and/or presence of uncollected garbage/refuse inside the house compounds were significantly associated with acute diarrhea.

The odds of developing acute diarrhea in households that shared one latrine among six or more households were 4.7 times (AOR = 4.7; 95% CI: 2.4–9.4) higher than for those sharing one latrine among one to five households. The odds of developing acute diarrhea in households with proximity of latrines within 15 meters were 6.6 times (AOR = 6.6; 95% CI: 2.5–17.0) higher than in households having latrines farther away. The likelihood of children developing acute diarrhea where feces were observed on the floor and/or around the sanitation facilities was 3.9 times (AOR = 3.9; 95% CI: 1.5–10.3) higher than for children having clean latrines. Furthermore, the likelihood of children developing acute diarrhea where flies were observed on the floor and/or around the sanitation facilities was 2.5 times (AOR = 2.5; 95% CI: 1.3–5.0) higher than for children having sanitation facilities where no flies were observed. The likelihood of children developing acute diarrhea in households having uncollected garbage/refuse observed inside the house compound was 3.2 times (AOR = 3.2; 95% CI: 1.2–8.4) higher than for those in households where refuse was regularly collected (Table 3).

**Table 3:** Sanitation facility and hygiene factors independently associated with acute diarrhea in multivariable logistic regression analysis*^£^.

Variable	AOR (95% CI) ^a^
Six or more households sharing one sanitation facility	4.7(2.4–9.4)
Proximity of sanitation facilities to home (< 15 meters)	6.6(2.5–17.0)
Feces observed on the floor and/or around the sanitation facilities	3.9(1.5–10.3)
Flies observed on the floor and/or around the sanitation facilities	2.5(1.3–5.0)
Uncollected garbage/refuse observed inside the house compound	3.2(1.2–8.4)

AOR, Adjusted odds ratio; CI, Confidence interval.

^a^Denotes adjusted odds ratio using 95% confidence interval in multivariable logistic regression analysis.

*Variables included in the multivariable analysis were number of households sharing one sanitation facility, proximity of sanitation facility to homes, feces and flies observed on the floor and/or around the sanitation facilities, uncollected garbage/refuse and domestic sewage observed inside the house compound, garbage/refuse disposal methods, and domestic sewage discharge methods.

^**£**^Socio-demographic factors included in the multivariable analysis were slum district; age, education, and marital status of caregivers; monthly household income; household size; house ownership; child’s age and sex.

## Discussion

This community-based cross-sectional study examined the relationship between acute diarrhea in children aged 0–50 months and sanitation and hygiene practices in slum areas of Addis Ababa. We found that prevalence of acute diarrhea was 11.9% and that most slum households used unimproved sanitation facilities, including public latrines, pit latrines without a slab, and open defecation. Acute diarrhea was significantly associated with presence of feces and flies on the floor and /or around the sanitation facilities, shared use of sanitation facilities by six or more households, proximity of latrines within 15 meters of homes, and the presence of uncollected garbage/refuse inside the house compound.

The acute diarrhea prevalence in our study is similar to the 12% prevalence of diarrhea reported by the Demographic and Health Survey for Ethiopia in 2016 [35]. However, whereas the overall diarrhea prevalence for Ethiopia was reported from aggregated data, the acute diarrhea statistic in our study was to a large extent due to poor sanitary facilities and poor hygienic practices. Studies in slums of Kenya [36], Rwanda [37], India [38, 39], and Nepal [40] reported higher diarrhea prevalence, apparently due to even poorer sanitation and hygiene status than in the Addis Ababa slums. The relatively lower prevalence of acute diarrhea in our study compared to the rates in the other slum areas mentioned might be due to the implementation of urban health extension programs by the Ethiopian government; these programs focus on improving sanitation and hygiene conditions in rural and urban areas, including slums, through the use of urban health extension workers. The active involvement of health professionals in hygiene and sanitation is crucial to accelerating and consolidating progress in disease prevention [41].

Our study showed that widespread sharing of sanitation facilities by households was associated with acute diarrhea. The widespread sharing of sanitation facilities among six or more households in Addis Ababa slums appears to be due primarily to lack of space for the construction of private latrines. A study in the Kibera slums in Nairobi in 2010 found that respondents used public latrines due to scarcity of private household latrines and the poor condition of other existing sanitation facilities in that crowded area [6]. Consistent with our findings, several other studies showed that sharing of sanitation facilities was associated with diarrhea [42, 43]. Heijenen et al. [42, 44] reported that households sharing sanitation facilities were generally poorer than those that did not share, and also had an increased risk for diarrhea, not necessarily because of sharing sanitation facilities, but because of poverty. In contrast to our findings, Demographic and Health Survey data covering 51 countries between 2001 and 2011 indicated that sharing sanitation facilities was a protective factor in diarrhea among under-five children, particularly in Nigeria, Mali, Senegal, and Liberia [45]. Baker et al. did not find that sharing of sanitation facilities in Bangladesh posed higher risks of diarrhea [43]. In our study, the indication that the sharing of sanitation facilities is a major concern may be due to a higher number of households (six or more) sharing one sanitation facility, a higher number of people per household, the predominance of unimproved sanitation facilities, and widespread sharing of both unimproved and improved sanitation facilities. The number of users per shared sanitation facility tends to be inversely related to the cleanliness of the sanitation facilities [12, 46].

The proportion of households practicing open defecation in our study was relatively low and not a risk factor in acute diarrhea. A higher rate (11%) of open defecation was reported in eastern Ethiopia [47]. The open defecation practice in our study was self-reported, indicating questionable reliability of the data. Another study in Ethiopia revealed that open defecation practice was underreported [18]. In contrast to our findings, a multicenter study in Kenya reported that practicing open defecation was a risk factor for moderate to severe diarrhea [43]. This discrepancy might be due to the low proportion of households practicing open defecation in Addis Ababa slum areas. Our finding is supported by previous studies in slums of Addis Ababa showing similar rates of open defecation [48]. Bartlett [49] found that the lack of latrines in poor communities causes many people to defecate in the open or into plastic bags and papers that are then discarded with the household garbage. The practice of open defecation in slums of Addis Ababa appears to be due largely to a combination of lack of latrine access; unusable, overflowing latrines; and poor hygienic conditions of shared latrines. A recent study in eastern Ethiopia found a lack of effective social mobilization to be the main cause for open defecation [47].

We also found that the presence of flies and feces on the floor and/or around the sanitation facilities was significantly associated with acute diarrhea. The presence of flies on the floor and/or around sanitation facilities appears to be due to poor cleanliness of the sanitation facilities, disposal of garbage close to sanitation facilities and inside the housing compound, and discharge of domestic sewage around the sanitation facilities and inside the housing compound. The presence of interruptions to water supplies in slums of Addis Ababa [50] might also be an obstacle to the regular cleaning of latrines. The lack of cleanliness of the sanitation facilities and housing compounds in our study might arise from the unwillingness of users to clean shared (versus private) sanitation facilities and a lack of commitment to regular cleaning of the overcrowded general living environment. Another study revealed that shared sanitation facility users are not committed to cleaning shared latrines [51]. A consistent finding in the slums of Huye Town in Rwanda was that the presence of flies within and around sanitation facilities was significantly associated with increased odds of contracting diarrhea [37].

Another study found that the disposal of garbage close to homes was a significant risk factor for high fly densities and the presence of flies around the sanitation facilities was, in turn, associated with acute diarrhea [52]. Strina et al. [53] found in Salvador, Brazil, that people in latrine-owning households behaver more hygienically than those without latrines. Public latrines are unhygienic and characterized by the presence of flies and floors dirty with feces [37, 54]. A similar finding in Rajshahi City slums in Bangladesh revealed that 61% of the latrines had observable feces [55]. Unsanitary conditions of latrines and poor hygiene behavior were significantly associated with acute diarrhea episodes in slums of An-Nasr in Jordan, Tebbaneh in Libya [56], and Ikare-Akoko in Nigeria [57]. Findings that substandard latrine construction contributes to the presence of flies indicate that improved superstructures may decrease fly densities around latrines [58, 59].

This study also revealed that the presence of uncollected garbage inside the housing compounds was significantly associated with acute diarrhea, consistent with studies in the slum areas of Huye Town in Rwanda and Dhaka slums in Bangladesh [37, 60]. Sanitation problems are usually aggravated by inadequate waste management [23, 49], a situation also characteristic of the slums in Addis Ababa. A study in urban slums in southern India revealed that the majority (66.1%) of the households indiscriminately dumped garbage/refuse outside [61]. The lower proportion of waste dumping in our study might be due to the urban health extension programs in Addis Ababa headed by health extension workers, who advocate proper disposal of garbage/refuse. Other studies in Ethiopia revealed that households following the recommendations of health extension workers showed lower diarrhea prevalence rates [62, 63] and better performance in primary healthcare [64].

We found that proximity of sanitation facilities to homes was inversely associated with acute diarrhea. This may be due to increased risk of transmission of pathogens via flies. Studies in Bangladesh and Kenya also revealed that closer proximity of latrines increased contamination of tube-well water sources and thereby contributed to diarrheal disease [65, 66]. Increased housing density in Addis Ababa in general, and in the slums in particular, as a result of rapid urbanization in recent years tended to decrease the distance between houses and latrines.

### Limitations of the study and gaps for future research

Our findings should be interpreted in light of certain limitations. First, although we used an adjusted multivariable logistic regression model, statistical adjustment can control for measured confounders but not for other complex confounding covariates that were not measured. Therefore, because there is residual confounding due to unmeasured variables, further studies are recommended that consider sanitation facility and hygiene variables that were not included in this study.

Another limitation is that our study was not conducted in the rainy season. During this time, diarrhea incidence tends to peak [67] and unsealed latrines in slums may overflow and disperse pathogens [68], although a study by Mukabutera et al. reported high diarrhea rates during the dry season [69]. Furthermore, cleaning of shared sanitation facilities in slums is impacted by the wet season, which worsens the already bad sanitation situation [70], as indicated by the seasonal trend of diarrhea cases [71]. Therefore, we encourage further studies to investigate the cleanliness of shared sanitation facilities and factors associated with poor hygienic practices in shared sanitation facilities during the rainy seasons in the slums of Addis Ababa and other urban slums in Ethiopia. Such studies may lead to comprehensive measures that can help to reduce acute diarrhea.

Various studies indicate that malnutrition increases the risk of diarrhea [72, 73] and that open defecation and heavy diarrhea burden increase the risk of stunting [60, 74, 75]. Children exposed over time to poor sanitation and poor hygiene may develop environmental enteropathy (tropical enteropathy), which is implicated as a cause of malnutrition [76–79]. Therefore, further studies that explore the linkage between environmental enteropathy and malnutrition and the effect of malnutrition on diarrhea (and vice versa) among under-five children in slum areas of Addis Ababa are also encouraged.

## Conclusion

Shared use of sanitation facilities, poor cleanliness of sanitation facilities, proximity of sanitation facilities to homes, and indiscriminate dumping of garbage/refuse inside house compounds were independently associated with acute diarrhea. Public health measures, such as further improvements of the existing municipal garbage/refuse collection and disposal system, construction of more improved sanitation facilities, and intensification of sanitation and hygiene promotion programs at the district and household levels may improve the cleanliness of the sanitation facilities and housing compounds and thereby reduce the risk of acute diarrhea among under-five children.

The slum renewal programs that have been in progress since 2005 in Addis Ababa [2] and the Urban Safety Net Program enacted in January 2017 by the Ethiopian government to enhance the livelihoods of poor urban residents [80] together with urban WASH (water, sanitation, and hygiene) [81] and the urban health extension [82] programs, are encouraging steps towards sustainable improvements in the cleanliness of slum sanitation facilities and housing compounds. These programs may achieve significant improvement in the sanitation facilities and hygiene status of the slums if they are carried out within an integrated framework that also addresses the livelihoods of the predominantly poor population and if interventions are monitored comprehensively and outcomes evaluated.

## Supporting information

S1 File Sanitation facilities and hygienic conditions data. (DTA)

## Abbreviations

AOR: Adjusted odds ratio
CI: Confidence interval
JMP: Joint Monitoring Programme
OR: Crude odds ratio
UNICEF: United Nations Children's Fund
WHO: World Health Organization
